# Optimising the extraction rate of a non-durable non-renewable resource in a monopolistic market: a mathematical programming approach

**DOI:** 10.1186/s40064-015-1276-0

**Published:** 2015-09-17

**Authors:** Albert Corominas, Enric Fossas

**Affiliations:** Institute of Industrial and Control Engineering, Universitat Politècnica de Catalunya, Av. Diagonal, 647, 08028 Barcelona, Spain

**Keywords:** Non-renewable resources, Optimal extraction rate, Non-linear programming

## Abstract

We assume a monopolistic market for a non-durable non-renewable resource such as crude oil, phosphates or fossil water. Stating the problem of obtaining optimal policies on extraction and pricing of the resource as a non-linear program allows general conclusions to be drawn under diverse assumptions about the demand curve, discount rates and length of the planning horizon. We compare the results with some common beliefs about the pace of exhaustion of this kind of resources.

## Background

Some natural resources are renewable (e.g. forests or fish stocks) whereas others are non-renewable (all minerals). Among the latter, some are durable (such as gemstones, precious metals and other metals like copper) whereas others are not (e.g. fossil fuels, phosphates and fossil water).

Durable non-renewable resources may be reused after consumption. Therefore, at any time there is an inventory of the resource in the ground and an inventory of the already used amounts of the resource that are potentially reusable. Conversely, non-durable non-renewable resources disappear as such when they are used (burnt or dispersed). This paper deals with the latter.

Most economic theory is not explicit about whether inputs into production are renewable or non-renewable. This is probably because until the second half of the twentieth century the idea prevailed that the Earth had plenty of resources relative to human needs. However, for more than 50 years it has been developing a growing awareness that the quantity of certain resources is limited, taking into account the past and present rates of consumption rates. Establishing optimal extraction and pricing policies for these resources requires specific approaches.

However, the first paper to explicitly consider the assumption of exhaustibility was Gray (1914), which shows that the present value of the marginal net revenue obtained from an exhaustive resource must be the same in all periods with positive extraction (Conrad 2010). In Hotelling (1931), the author wrote as the introduction and motivation of his paper that “Contemplation of the world’s disappearing supplies of minerals, forests, and other exhaustible assets has led to demands for regulation of their exploitation. The feeling that these products are now too cheap for the good of future generations, that they are being selfishly exploited at too rapid a rate, and that in consequence of their excessive cheapness they are being produced and consumed wastefully has given rise to the conservation movement.”

Since Hotelling’s paper, many works have been published about the economy of exhaustible resources (see overview in Sect. “Literature review”). Nonetheless, many questions concerning their optimal extraction and pricing policies under a variety of specific assumptions remain unanswered. The present paper deals with the determination of optimal policies for a monopolist of a non-durable non-renewable resource using non-linear programming and Karush, Kuhn and Tucker (KKT) conditions. Although mathematical programming typically yields only numerical results for each instance of a given problem, in the case in point the specific simple structure of the resulting non-linear programs makes it possible, and this is the contribution of the paper, to draw conclusions about the shape of optimal policies under diverse assumptions concerning demand curves, discount rates, costs (including scenarios in which these three elements are time-dependent) and lengths of the planning horizon.

The rest of the paper is organised as follows. The next section is devoted to a brief literature review. Section “Problem definition and formulation of mathematical programming models” states the problem and formulates the corresponding mathematical programs. The properties of the optimal solutions are discussed in Sect. “Properties of the optimal solutions”, which also contains some numerical examples. Section “Conclusions” closes the paper with some concluding remarks and future research lines.

## Literature review

There is abundant literature on the determination of optimal policies for extracting and pricing exhaustible resources.

Leaving aside the precursor paper of Gray, there is a consensus (Solow 1974; Devarajan and Fisher 1981; Arrow 1987; Gaudet 2007) on the unique, seminal character of Hotelling (1931) regarding the economic theory of non-renewable resources. This work discusses the dynamics of optimal prices and production rates of irreplaceable assets under the assumptions of free competition, monopoly and duopoly. The author points out that the use of the calculus of variations (Pontryagin et al. 1986) cannot be avoided in dealing with problems concerning exhaustible assets. As this technique was not widespread at that time, the Economic Journal rejected Hotelling’s paper because its mathematics was too difficult (Gaudet 2007).

The nowadays famous Hotelling’s rule is stated in Hotelling (1931) in a very simple way: “It is not unreasonable to expect that the price [of an exhaustible resource] will be a function of the time of the form $p = p_{0} \cdot \exp \left( {\gamma t} \right)$” where $\gamma$ is “the force of interest”. Hotelling points out, however, that this “is characteristic of completely free competition” and “will not apply to monopoly, where the form of the demand function is bound to affect the rate of production”. Moreover, in this latter case, “if the demand curve is fixed, the question whether the time until exhaustion will be finite or infinite turns upon whether a finite or infinite value of *p* will be required to make *q* vanish. For the demand function *q* = exp(−*bp*), where *b* is a constant, the exploitation will continue forever, though of course at a gradually diminishing rate. If $q = \alpha - \beta p$, all will be exhausted in a finite time.” Therefore, Hotelling’s rule is not an attempt to describe the behaviour of the real world, but an implication of assuming completely free competition.

After Hotelling’s paper, the rate of publication on the subject was low until the seventies of the past century. Byé (1957), which examines the case of the firm that decides the period over which the operation of a given natural resource is spread out, and Barnett and Morse (1963), which minimises the relevance of scarcity, are two significant references from that period.

Concerns about natural resource scarcity were triggered by the famous report *Limits to Growth* by the Club of Rome (Meadows et al. 1972) and the 1973 oil crisis. At the Eighty-Sixth Annual Meeting of the American Economic Association, held in New York, December 1973, Solow gave the Richard T. Ely Lecture on the economics of resources (Solow 1974), in which he highlighted the relevance and contemporaneousness of Hotelling’s paper. Since those days, the number of publications about the economy of exhaustible resources has increased steadily.

The comparison between the rates of production of an exhaustible resource in competitive and monopolist markets, already dealt with in Hotelling (1931), is also addressed in Stiglitz (1976).

Many papers discuss or try to improve and test Hotelling’s rule, e.g. Kay and Mirrlees (1975), Riley (1980), Slade (1982), Slade and Thille (1997), Livernois and Martin (2001), Livernois et al. (2006), Gaudet (2007), Livernois (2009) and Slade and Thille (2009).

Others focus on extraction costs, e.g. Heal (1976), Gilbert (1978), Hanson (1980), Farzin (1992, 1995) and Krautkraemer (1998). Gaudet et al. (2001) addresses specifically transportation costs when resource sites are spatially distributed.

Uncertainties in the amount of the resource available and the related question of exploration and investment to find new deposits are discussed in Long (1975), Loury (1978), Pindyck (1978), Arrow and Chang (1982), Liu and Sutinen (1982), Gaudet and Khadr (1991) and Ghoddusi (2010).

The concept of peak oil, also known as Hubbert’s peak, was introduced in Hubbert (1956). The Hubbert curve resulted from fitting historic production data to a symmetric bell-shaped function (Cleveland and Kaufmann 1991).Therefore, initially it was an empirically based result. Subsequently, other authors have come up with economic explanations of why the Hubbert curve or similarly shaped curves represent the time evolution of non-durable non-renewable resource production (Menard and Sharman 1975; Cleveland and Kaufmann 1991; Al-Jarri and Startzman 1999; Reynolds 1999; Bardi 2005, 2007; Jakobsson et al. 2012). In short, the basic element of the proposed models is the fact that these resources have unknown reserves that should be explored. Recently, debates over whether Hubbert’s peak has been reached have become frequent. From an analysis of global production data, Aleklett et al. (2010) concludes that the peak oil was probably reached in 2008 and Campbell (2012) holds a similar point of view. On the other hand, Lynch (2003) does not give validity to the theory of peak oil itself.

Comprehensive treatments of the economy of exhaustible resources may be found in Dasgupta and Heal (1979), Conrad and Clark (1987), Sweeney (1993), Conrad (2010) and Perman et al. (2011).

Following Hotelling’s approach, time is commonly regarded as a continuous variable. A proposal to use mathematical programming to determine optimal policies on exhaustible resources considering discrete time was suggested, but not developed, in Conrad and Clark (1987). Sweeney (1993) and Conrad (2010) also consider the discrete time case for competitive and monopolistic markets using a Lagrangian approach.

Some publications dealing with optimal policies of exhaustible resources in a monopolistic market contain the formulation of the optimisation problem, the necessary and sufficient conditions of optimality and the solution to particular cases, such as linear demand curve. However, the general properties and conclusions we derive using a non-linear programming formulation and KKT conditions under diverse assumptions about the demand curve, discount rates and length of the planning horizon are new, at the best of our knowledge.

## Problem definition and formulation of mathematical programming models

We deal with a monopolistic market of a non-durable non-renewable resource whose available amount, $R$, is assumed to be positive and known. The planning horizon is divided into $T$ periods of equal (or different) length.

The demand curve of each period, $t$, is also assumed known by means of a function giving the quantity, $q_{t}$, from the price, $p$, considered as the argument, or the inverse demand curve, giving the price from the quantity considered as the argument. Therefore, we have either $q = \text{q}_{t} \left( p \right)$ or $p = \text{p}_{t} \left( q \right)$, which we assume to be differentiable and having the following properties:

$$ \begin{aligned} \text{q}_{t} \left( p \right) & \ge 0,\,\,\quad \frac{{d{\text{q}}_{t} \left( p \right)}}{dp} < 0,\,\,\quad 0 \le p \le P_{t} ,\,\quad {\text{where}}\,\,P_{t} |\text{q}_{t} \left( {P_{{_{t} }} } \right) = 0 \\ {\text{p}}_{t} \left( q \right) & \ge 0,\,\,\quad \frac{{d{\text{p}}_{t} \left( q \right)}}{dq} < 0,\,\quad \,0 \le q \le Q_{t} ,\,\quad \,{\text{where}}\,\,Q_{t} |{\text{p}}_{t} \left( {Q_{{_{t} }} } \right) = 0 \\ \end{aligned} $$

$P_{t} \,$ is called the choke price, which, for some kinds of demand curves, is not bounded above.

Moreover, we assume:

- The cost of extracting a unit of resource, $c_{t} \,\left( {t = 1, \ldots ,T} \right)$, depends on the period and is also known. We also assume that $c_{t} < P_{t} \,\,\left( {t = 1, \ldots ,T} \right)$, since otherwise there would be no extraction at all during period $t$.
- The discount coefficients $\alpha_{t} \,\left( {t = 1, \ldots ,T} \right)\,$ to be applied to the cash flows of each period to obtain their present values are known.
- The full amount of resource extracted in a period must be consumed during that period (i.e. there is no possibility to stockpile the resource during a period for sale in subsequent periods).

Then, we can formulate the following non-linear mathematical program (MQ) in order to obtain the optimal extraction policy:

$$ \begin{aligned} & {\text{MQ}} \\ & maximise\,\quad \sum\limits_{t = 1}^{T} {\alpha_{t} \cdot \left[ {{\text{p}}_{t} \left( {q_{t} } \right) - c_{t} } \right]} \cdot q_{t} \, \\ & {\text{s}} . {\text{t}} .\\ \end{aligned} $$

1 $$\begin{aligned}  \sum\limits_{t = 1}^{T} q_{t} & \le R  \end{aligned}$$

2 $$ \, - q_{t} \le 0 $$

3 $$ q_{t} \le Q_{t} $$

Thus, it is clear that the problem of finding the optimal extraction policy for a non-durable non-renewable resource is not much different from that of maximising the utility of a consumer having a given amount of income to spend, which, as it is known, leads to Gossen’s second law. According to it, the ratio of the marginal utility of each good or service to its price has the same value for all effectively consumed products and it is not less than the ratios corresponding to non-consumed products.

Note that if, instead of quantities, prices are used as variables, a similar mathematical program can be formulated in which the objective function and also the constraint corresponding to the availability of the resource are non-linear. Therefore, MQ is simpler, because all its constraints are linear, and is the only that will be used in the rest of the paper.

## Properties of the optimal solutions

For $R > 0$, MQ has a non-empty set of feasible solutions with interior points.

Therefore, Karush, Kuhn and Tucker conditions are necessary and sufficient for optimality if $\text{p}_{t} \left( {q_{t} } \right) \cdot q_{t}$ is concave $\forall t$. In what follows, we will assume that this property holds.

For instance,

- $$ {\text{If}}\;\,q = 1 - p^{\gamma } \;\,{\text{with}}\;\,\gamma \ge 1,\,\;{\text{then}}\,\;\,{\text{p}}\left( q \right) \cdot q\;\,{\text{is}}\;\,{\text{concave}} . $$
- $$ {\text{If}}\,\,q = e^{ - p} \,{\text{then}}\,\,{\text{p}}\left( q \right) \cdot q\,\,{\text{is}}\,{\text{concave}} . $$

We will apply the Karush, Kuhn and Tucker conditions to find the optimal solutions of the non-linear program MQ. Let $u_{o}$ be the multiplier corresponding to constraint (1) and $u_{1} , \ldots ,u_{T}$ those associated with constraints (2).

Recall that $\frac{{d{\text{p}}_{t} \left( q \right)}}{dq} \le 0\,\,\forall t$ and $\frac{{d^{2} \left[ {{\text{p}}_{t} \left( q \right) \cdot q} \right]}}{{dq^{2} }} \le 0\,\,\forall t$ (because of the concavity of $\text{p}_{t} \left( {q_{t} } \right) \cdot q_{t}$). If we assume that $\left( {\,\frac{{d{\text{p}}_{t} \left( q \right)}}{dq}} \right)_{q = 0}$ has an upper bound or that ${ \lim }_{q \to 0} \, \frac{{d{\text{p}}_{t} \left( q \right)}}{dq} \cdot q = 0$, then holds the following:

### Proposition

$$ \left( {q_{t}^{*} > 0 \wedge q_{{t^{'} }}^{*} = 0} \right) \Rightarrow \left( {\alpha_{t}  \cdot \hat{P}_{t} \ge \alpha_{{t^{'} }}  \cdot \hat{P}_{{t^{'} }} } \right) $$

where $t$ and $t'$ are two periods belonging to the planning horizon, $q_{t}^{*}$ and $q_{t'}^{*}$ the corresponding quantities in an optimal policy and $\hat{P}_{t} = P_{t} - c_{t} ,\,\,\hat{P}_{t'} = P_{t'} - c_{t'}$ (net choke prices). The proof of the proposition is shown in “Appendix 1”.

Hence, if the periods are arranged in the non-increasing order of the products $\alpha_{t}  \cdot \hat{P}_{t} ,$ there is always an optimal policy such that the periods in which $q_{t}^{*} > 0$ are either all those belonging to the planning horizon or a certain number of them occupying the first positions in the defined sequence. Notice that the order of the periods depends neither on the available amount of the resource nor on the shape of the demand curves.

This property makes it possible to easily determine the optimal policy under diverse assumptions.

Let us first suppose that *T* is too short to allow for exhaustion of the resource when the optimal policy is used.

Then,

$$ \begin{aligned} & \sum\limits_{t = 1}^{T} {q_{t}^{*} } < R\,\, \Rightarrow u_{0} = 0 \\ & {\text{p}}_{t} \left( {q_{t}^{*} } \right) - c_{t} + q_{t}^{*}  \cdot \left( {\frac{{d{\text{p}}_{t} \left( q \right)}}{dq}} \right)_{{q = q_{t}^{*} }} = 0\, \\ & \quad \forall t \in T|q_{t}^{*} > 0 \\ \end{aligned} $$

i.e. the optimal quantities depend neither on R nor on the discount coefficients (therefore, nor on the interest rates).

E.g.

$$ \begin{aligned} & {\text{For}}\;{\text{the}}\;\,{\text{function}}\,\;\,{\text{p}}_{t} \left( q \right) = P_{t} \cdot\left( {1 - \frac{q}{{Q_{t} }}} \right)\,\;{\text{we}}\;\,{\text{obtain }}\;q_{t}^{*} = \frac{{Q_{t} }}{{2 \cdot P_{t} }} \cdot \hat{P}_{t} \,\;{\text{and}}\,\;p_{t}^{*} = \frac{1}{2} \cdot \left( {P_{t} + c_{t} } \right).\; \\ \, & {\text{For}}\;\,{\text{p}}_{t} \left( q \right) = - \frac{1}{{\lambda_{t} }} \cdot \ln \frac{{q_{t} }}{{Q_{t} }},\quad q_{t}^{*} = \frac{{Q_{t} }}{e} \cdot e^{{ - \lambda_{t}  \cdot c_{t} }} \;\,{\text{and}}\,\;\,p_{t}^{*} = \frac{1}{{\lambda_{t} }} + c_{t} . \\ \end{aligned} $$

Let us call these optimal quantities, $$ \overset{\lower0.5em\hbox{$\smash{\scriptscriptstyle\smile}$}}{q}_{t}^{*} $$, stock-independent optima.

Thus, when the planning horizon is too short for the sum of stock-independent optima to be greater than *R,* the optimal behaviour of the monopolist is the same as if the stock of the resource were unlimited.

Let $$\mathop{T}\limits_{}^{\smile}$$ be the maximum value of $$ T $$ such that $$ \sum\nolimits_{t = 1}^{T} {\overset{\lower0.5em\hbox{$\smash{\scriptscriptstyle\smile}$}}{q}_{t}^{*} } \le R\, $$.

Then, when $$ T > \mathop{T}\limits_{}^{\smile} $$ the resource will be exhausted. If we call active a period in which the extraction is not null, and we arrange the periods in the planning horizon in the non-increasing order of $$ \,\alpha_{t}  \cdot \hat{P}_{t} $$, all or only a certain number of those periods that occupy the first positions in the above order will be active. We will use the notation [*x*] to indicate the period that occupies the position *x*. The number of active periods ($$ \tau $$, such that $$ \overset{\lower0.5em\hbox{$\smash{\scriptscriptstyle\smile}$}}{T} \le \tau \le T $$) increases monotonically with $$ T $$ and, when the planning horizon is unlimited, may or may not have an upper bound.

The condition $u_{0} = \alpha_{\left[ t \right]}  \cdot \left( {\left( {\frac{{d\left[ {{\text{p}}_{t} \left( q \right) \cdot q} \right]}}{dq}} \right)_{{q = q_{\left[ t \right]}^{*} }} - c_{t} } \right)$ must hold for $t = 1, \ldots ,\tau$ and must not for ${\text{t}} > \tau$ (when $\tau < {\text{T}}$). The right-hand side depends only on $q_{\left[ t \right]}^{*}$, so we may write $u_{0} = \upvarphi_{\left[ t \right]} \left( {q_{\left[ t \right]}^{*} } \right)$. If $\text{p}_{\left[ t \right]} \left( q \right) \cdot q$ is strictly concave, then $\upvarphi_{\left[ t \right]} \,$ is strictly decreasing. Therefore, $q_{\left[ t \right]}^{*} = \upvarphi_{\left[ t \right]}^{ - 1} \left( {u_{0} } \right)$, which is also strictly decreasing. Hence, so is $\sum\nolimits_{t = 1}^{\tau } {\upvarphi_{\left[ t \right]}^{ - 1} \left( {u_{0} } \right)}.$ Thus, the equation $\sum\nolimits_{t = 1}^{\tau } {\upvarphi_{\left[ t \right]}^{ - 1} \left( {u_{0} } \right)} = R$ has a unique solution $u_{0} = \Phi \left( R \right) \ge 0$ and the function $\Phi \left( R \right)$ is therefore strictly decreasing (as expected, as $u_{0}$ is the shadow price of the resource; moreover, given $R$, $u_{0}$ increases monotonically with $T$ because the shadow price of the resource cannot decrease when the planning horizon increases, since this enlarges the space of feasible solutions). With the value $\Phi \left( R \right)$ we can obtain the optimal values of the quantities $q_{\left[ t \right]}^{*} = \upvarphi_{\left[ t \right]}^{ - 1} \left( {\Phi \left( R \right)} \right) \ge 0$.

Indeed, $\tau$ is not known a priori unless the products $\,\alpha_{t}  \cdot \hat{P}_{t}$ are unbounded $\left( {t = 1, \ldots ,T} \right),$ in which case $\tau = T$. In order to find its value, we can use either of the following two ways.

On the one hand, we can give increasing values to $\tau$, beginning with $\tau =\mathop{T}\limits_{}^{\smile}$ and stopping when the optimality condition does not hold or, of course, when $\tau = T$.

On the other hand, when it has a finite upper bound, $\tau$ depends on the amount of the resource available, $R$. If we call $r(n)$ the critical value for which the period $\left[ n \right]$ becomes active, the following conditions must hold:

$$ \begin{aligned} & \sum\limits_{t = 1}^{n - 1} {q_{\left[ t \right]}^{*} } = r(n) \\ & \alpha_{\left[ t \right]} \cdot \left( {\left( {\frac{{d\left[ {{\text{p}}_{\left[ t \right]} \left( q \right) \cdot q} \right]}}{dq}} \right)_{{q = q_{\left[ t \right]}^{*} }} - c_{\left[ t \right]} } \right) = \alpha_{\left[ n \right]} \cdot\left( {\left( {\frac{{d\left[ {{\text{p}}_{\left[ n \right]} \left( q \right) \cdot q} \right]}}{dq}} \right)_{q = 0} \, - c_{\left[ n \right]} } \right)\, = \alpha_{\left[ n \right]}  \cdot \hat{P}_{\left[ n \right]} \,\,\, \\ & t = 1, \ldots ,n - 1 \\ \end{aligned} $$

Given the values of $r(n)$, it is straightforward to find $\tau$ for any specific $R$.

Let us see two examples. In both we assume $T > \mathop{T}\limits_{}^{\smile}$.

### *Example 1*

$$ {\text{p}}_{t} \left( q \right) = P_{t} \cdot\left( {1 - \frac{q}{{Q_{t} }}} \right). $$

From KKT, we have

$$ q_{\left[ t \right]}^{*} = \frac{{Q_{\left[ t \right]} }}{{2 \cdot P_{\left[ t \right]} }} \cdot \left( {\hat{P}_{\left[ t \right]} - \frac{{u_{0} }}{{\alpha_{\left[ t \right]} }}} \right)\,\forall t \le \tau $$

Then,

$$ u_{0} \left( \tau \right) = \frac{{\sum\nolimits_{t = 1}^{\tau } {\frac{{Q_{\left[ t \right]} }}{{P_{\left[ t \right]} }}}  \cdot \hat{P}_{\left[ t \right]} - 2 \cdot R}}{{\sum\nolimits_{t = 1}^{\tau } {\frac{1}{{\alpha_{\left[ t \right]} }}\frac{{Q_{\left[ t \right]} }}{{P_{\left[ t \right]} }}} }},\,\,\quad u_{0} \left( \tau \right) \ge 0\,\, $$

However, the condition $\alpha_{{\left[ {\tau + 1} \right]}}  \cdot \hat{P}_{{\left[ {\tau + 1} \right]}} \le u_{0} \left( \tau \right)\,$ must hold since

$$ \alpha_{{\left[ {\tau + 1} \right]}}  \cdot \hat{P}_{{\left[ {\tau + 1} \right]}} > u_{0} \left( \tau \right)\, \Leftrightarrow \,\left[ {u_{0} \left( {\tau + 1} \right)\, > u_{0} \left( \tau \right)} \right] \wedge \left[ {\alpha_{{\left[ {\tau + 1} \right]}}  \cdot \hat{P}_{{\left[ {\tau + 1} \right]}} > u_{0} \left( {\tau + 1} \right)} \right], $$

which would imply that $q_{{\left[ {\tau + 1} \right]}}^{*} > 0$ (contrary to the assumption that $\tau$ is the number of active periods). Therefore, to find $\tau$ it suffices to calculate the successive values $u_{0} \left( 1 \right),\,u_{0} \left( 2 \right) \ldots \tau$ is the first value fulfilling the condition $\alpha_{{\left[ {\tau + 1} \right]}} \cdot\hat{P}_{{\left[ {\tau + 1} \right]}} \le u_{0} \left( \tau \right)\,$; if there is no such value, then $\tau = T$.

### *Example 2*

$$ {\text{p}}_{t} \left( q \right) = - \frac{1}{{\lambda_{t} }} \cdot \ln \frac{{q_{t} }}{{Q_{t} }}. $$

From KKT,

$$ \begin{aligned} q_{t} &= \frac{{Q_{t} }}{e} \cdot \frac{{e^{{ - \lambda_{t}  \cdot c_{t} }} }}{{e^{{\frac{{u_{0} }}{{\alpha_{t} }}}} }} > 0 \\ \sum\limits_{t = 1}^{T} {q_{t} } &= \frac{1}{e}\sum\limits_{t = 1}^{T} {Q_{t}  \cdot \frac{{e^{{ - \lambda_{t}  \cdot c_{t} }} }}{{e^{{\frac{{u_{0} }}{{\alpha_{t} }}}} }}} = R \Rightarrow \quad\left [ {u_{o} > 0} \right] \wedge \left[ {u_{0} \left( {T + 1} \right) > u_{0} \left( T \right)} \right]\end{aligned}$$

All periods are active and the greater *T* is, the greater the profit.

Table 1 shows some significant features of the optimal policies for different combinations of demand curves, amounts of the resource available, discount coefficients and planning horizons.

**Table 1:** Some significant features of the optimal policies for different combinations of demand curves, amounts of the resource available, discount coefficients and planning horizons

Demand curve	$R$	$\alpha_{t}$	$T$	$q_{t}^{*}$	$p_{t}^{*}$	Annual income, $I_{t}$	$T$ min for depletion
$Q \cdot e^{ - \lambda \cdot p}$	$R$	$1\,\,\forall t$	$$ \begin{aligned} T \ge \hfill \\ R\cdot\frac{e}{Q} \hfill \\ \end{aligned} $$	$R/T$	$- \frac{1}{\lambda }\cdot\ln \frac{1}{T}$	$- \frac{R}{T}\cdot\frac{1}{\lambda }\cdot\ln \frac{1}{T}$	$\left\lceil {R\cdot\frac{e}{Q}} \right\rceil$
$150 \cdot e^{ - p}$	180	$0.95^{t - 1}$	10	$q_{10}^{*} /q_{1}^{*} = 0.59$	$p_{10}^{*} /p_{1}^{*} = 1.28$	$I_{10}^{*} /I_{1}^{*} = 0.76$	4
$q_{t} = 10,000 \cdot \left( {1 + \frac{t - 1}{4}} \right)\cdot\left( {1 - \frac{p}{{1000\cdot\left( {1 + \frac{t - 1}{4}} \right)}}} \right)$	18,000	$0.875^{t - 1}$	>11	$$ \begin{aligned} > 0\,\,\forall t \le 11 \hfill \\ = 0\,\,\forall t > 11 \hfill \\ \end{aligned} $$			3
			11	Max at $t = 6$ $q_{11}^{*} /q_{1}^{*} = 0.68$	Min at t = 2 $p_{11}^{*} /p_{1}^{*} = 2.91$	$I_{11}^{*} /I_{1}^{*} = 1.98$	
		$0.9^{t - 1}$	≤8	$> 0\,\forall t$			
			>9	$$ \begin{aligned} > 0\,\,\forall t|2 \le t \le 13 \hfill \\ = 0\,\,\forall t > 13 \hfill \\ \end{aligned} $$			
			13	Max at $t = 8$ $q_{13}^{*} /q_{2}^{*} = 3.75$	$p_{13}^{*} /p_{2}^{*} = 3.19$	$I_{13}^{*} /I_{2}^{*} = 11.96$	
	1000	$0.9^{t - 1}$	13	$>0\,\,t = 7, \ldots ,9$ $q_{9}^{*} /q_{7}^{*} = 0.31$	$p_{9}^{*} /p_{7}^{*} = 1.22$	$I_{9}^{*} /I_{7}^{*} = 0.38$	1
$q_{t} = 10,000\cdot\left( {1 + \frac{t - 1}{100}} \right)\cdot\left( {1 - \frac{p}{{1000\cdot\left( {1 + \frac{t - 1}{100}} \right)}}} \right)$	18,000	$0.875^{t - 1}$	9	$q_{9}^{*} /q_{1}^{*} = 0.08$	$p_{9}^{*} /p_{1}^{*} = 1.54$	$I_{9}^{*} /I_{1}^{*} = 0.12$	4
		$0.999^{t - 1}$	11	$q_{11}^{*} /q_{1}^{*} = 1.33$	$p_{11}^{*} /p_{1}^{*} = 1.06$	$I_{11}^{*} /I_{1}^{*} = 1.41$	

In all cases it is assumed that $c_{t} = 0\,\,\forall t$

Note that one of the assumptions that define the problem dealt with in this paper is that the amount of the resource, $R$, is known from the intial instant. If we consider that there are reserves to be discovered, the problem becomes considerably more complicated and lies beyond the scope of this work, although it is a promising line of future research. All the same, a simple setting including an expectation of founding new reserves of the resource is presented in “Appendix 2'' in order to compare the behaviour of different approaches.

## Conclusions

Mathematical programming is a useful tool to analyse the optimisation of the extraction rate of a non-durable non-renewable resource provided that demand curves and extraction costs depend only on time and not on availability of the resource.

Under monopolistic conditions, the optimal extraction rate depends on the demand curve features, discount coefficients and length of the time horizon.

Given the demand curves and discount coefficients, the shape of the optimal policy depends on the length of the time horizon and may consist in:

- Extracting, in all periods of the planning horizon, the same amount of the resource that would be extracted if its availability were unlimited and leaving the remaining stock in the ground, or
- Depleting the resource by either
  - extracting in all periods of the planning horizon, or
  - extracting only in those periods occupying the first $\tau$ positions in the non-increasing order of $\alpha_{t}  \cdot \hat{P}_{t}$.

Under the stated assumptions, there is no reason to suppose, apart from particular cases, that there is a peak ore, and even less that the peak would coincide with a stock equal to the half of the initial stock.

Optimal policies for some combinations of planning horizons, demand curves and discount coefficients result in positive extraction rates for a finite number of periods and an abrupt interruption of the supply of the resource. This may be compatible with a steady increase of the extraction rate and a moderate or even no increase of price throughout the planning horizon.

Future extensions could include consideration of extraction costs depending on the available stock, perfect competitive markets, uncertainty about future availability of the resource and determination of optimal policies for durable non-renewable resources.

## Appendix 1: Proof of the proposition $\left( {q_{t}^{*} > 0 \wedge q_{t'}^{*} = 0} \right) \Rightarrow \left( {\alpha_{t} \cdot \hat{P}_{t} \ge \alpha_{t'} \cdot \hat{P}_{t'} } \right)$

Particularising KKT conditions for MQ yields

$$\begin{aligned}
 - \alpha_{t}  \cdot \left( {\left( {\frac{d [{\text{p}}_{t} ( q)
\cdot q ]}{dq}} \right)_{q = q_{t}^{*}} - c_{t} } \right) + u_{0}
& = - \alpha_{t'}  \cdot \left( {\left(
{\frac{d [ {{\text{p}}_{t'} ( q) \cdot q}  ]}{dq}}
\right)_{q = 0} - c_{t'} } \right) + u_{0} - u_{t'} = 0 \\
u_{0} &= \alpha_{t} \cdot\left( {\left( {\frac{{d [ {\text{p}}_{t}
\left( q \right) \cdot q ]}}{dq}} \right)_{{q = q_{t}^{*} }} - c_{t}
} \right) \\
& = \alpha_{t'}  \cdot \left( {\left( {\frac{{d [
{{\text{p}}_{t'} ( q  ) \cdot q} ]}}{dq}} \right)_{q = 0} - c_{t'} }
\right) + u_{t'} ,
\end{aligned}$$

which implies $$ \alpha_{t}  \cdot \left( {\left( {\frac{{d\left[ {{\text{p}}_{t} \left( q \right) \cdot q} \right]}}{dq}} \right)_{{q = q_{t}^{*} }} - c_{t} } \right) \ge \alpha_{t'}  \cdot \left( {\left( {\frac{{d\left[ {{\text{p}}_{t'} \left( q \right) \cdot q} \right]}}{dq}} \right)_{q = 0} - c_{t'} } \right) $$.

Moreover, because of the concavity of $$ {\text{p}}_{t} \left( q \right) \cdot q:\,\,\,\,\,\,\, $$$$ \,\left( {\frac{{d\left[ {{\text{p}}_{t} \left( q \right) \cdot q} \right]}}{dq}} \right)_{{q = q_{t}^{*} }} \le \left( {\frac{{d\left[ {{\text{p}}_{t} \left( q \right) \cdot q} \right]}}{dq}} \right)_{q = 0} = \left( {{\text{p}}_{t} \left( q \right) + \frac{{d{\text{p}}_{t} \left( q \right)}}{dq} \cdot q} \right)_{q = 0} = P_{t} . $$

Therefore, $$ \alpha_{t}  \cdot \left( {\left( {\frac{{d\left[ {{\text{p}}_{t} \left( q \right) \cdot q} \right]}}{dq}} \right)_{{q = q_{t}^{*} }} - c_{t} } \right) \, \le \alpha_{t}  \cdot \left( {P_{t} - c_{t} } \right) = \alpha_{t}  \cdot \hat{P}_{t} \,\,\,\,\,\,\,\, $$.

On the other hand, $\left( {\frac{{d\left[ {{\text{p}}_{t'} \left( q \right) \cdot q} \right]}}{dq}} \right)_{q = 0} = \left( {{\text{p}}_{t'} \left( q \right) + \frac{{d{\text{p}}_{t'} \left( q \right)}}{dq} \cdot q} \right)_{q = 0} = P_{t'}$

Consequently, $$ \alpha_{t'}  \cdot \left( {\left( {\frac{{d\left[ {{\text{p}}_{t'} \left( q \right) \cdot q} \right]}}{dq}} \right)_{q = 0} - c_{t'} } \right) = \alpha_{t'}  \cdot \left( {P_{t'} - c_{t'} } \right) = \alpha_{t'}  \cdot \hat{P}_{t'} $$.

Therefore, $\alpha_{t}  \cdot \hat{P}_{t} \ge \alpha_{t'}  \cdot \hat{P}_{t'}$, which proves the proposition.

## Appendix 2

The traditional theory of investment supposes that the decision-maker should decide what to do in the planning horizon in the initial instant using the information available at that moment. The option approach (Dixit and Pyndick, 1994), however, considers the possibility of delaying decisions until the moment in which new information is available to avoid potential sunk costs. These ideas can be applied to the management of renewable (Pindyck 2007) and non-renewable (Reynolds 2013) resources. In some circumstances, the expectation of having a large reserve available in the future with a high (or even not so high) probability can lead to the exploitation of presently available resources as if there were certainty about the future availability of large reserves.

To illustrate these ideas, let us present and solve the decision problem in a very simple setting: $p= 1- q$, $T = 2$, $\alpha = 1/1.1$, $R = 0.75$, costs are negligible; at the end of period 1, the result of an exploration process underway will be known: the result will be positive (i.e. a very large—in fact, 0.5 units would be more than enough—new amount of resource will be available) with a probability equal to $\pi$ and will be negative (no new reserves will be available) with a probability equal to $1 - \pi$.

In a more general setting, the optimal policy should be determined by dynamic programming. However, in the example it is straightforward to find the maximum of the expected income values, which, if we call $$ x $$$$ \left( {\text{0} \le \text{x} \le R} \right) $$ the quantity produced in the first period, is equal to

$$ \text{x } \cdot (1 -\text{x}) + \alpha  \cdot \pi  \cdot \frac{1}{4}\text{ + }\alpha  \cdot \left( {1 - \pi } \right) \cdot \left( {R - x} \right) \cdot \left[ {1 - \left( {R - x} \right)} \right], $$

which is optimal for $x^{*} = \hbox{min} \left( {R,\frac{1 + \alpha  \cdot (1 - \pi ) \cdot (2 \cdot R - 1)}{{2 \cdot \left[ {1 + \alpha  \cdot \left( {1 - \pi } \right)} \right]}}} \right)$.

Note that, in this example, $$ \overset{\lower0.5em\hbox{$\smash{\scriptscriptstyle\smile}$}}{q}_{1}^{*} = \overset{\lower0.5em\hbox{$\smash{\scriptscriptstyle\smile}$}}{q}_{2}^{*} = 0.5 $$. Hence, this is the optimal production value if the monopolist has a sufficient amount of the resource available.

Let us consider three different extraction policies:

P1: In the first period, we will act as if we were sure of having a large reserve available at the beginning of the second period. Therefore, we will extract $$ \overset{\lower0.5em\hbox{$\smash{\scriptscriptstyle\smile}$}}{q}_{1}^{*} = 0.5 $$ in the first period and, in the second, 0.5 or 0.25, according to the positive or negative result of the prospection, respectively
P2: In the first period, we will extract the quantity corresponding to the optimal policy under the assumption that we will have only the initially available $R$ units of the resource. Then, in the second period, according to the positive or negative result of the prospection, we will extract 0.5 or $R$ minus the production of the first period
P3: It is the optimal policy under the assumptions defining the example. In the first period, we will extract $x^{*}$ and, in the second, 0.5 or $R - x^{*}$, depending on the outcome of the exploration process

Table 2 shows the expected income values for several values of the probability $\pi$ corresponding to the three above policies.

**Table 2:** Expected income values corresponding to three extraction policies for different values of the probability $\pi$ of having a positive result of the prospection underway

$\pi$	0.0	0.1	0.2	0.3	0.4	0.5	0.6	0.7	0.8	0.9	1.0
P1	0.420	0.426	0.432	0.438	0.443	0.449	0.455	0.460	0.466	0.472	0.477
P2	0.444	0.445	0.447	0.449	0.451	0.453	0.455	0.457	0.459	0.461	0.463
P3	0.448	0.449	0.451	0.453	0.455	0.458	0.461	0.464	0.468	0.472	0.477

The differences between the best expected value (which always corresponds to P3) and the worst one are always lower than 7 %. P1 is better than P2 for $\pi < 0.6$ and worse for $\pi > 0.6$. Of course, when $\pi = 1.0$, P1 and P3 are equivalent.
